# The impact on body image and quality of life in breast cancer patients

**DOI:** 10.1590/0034-7167-2024-0207

**Published:** 2025-03-10

**Authors:** Andressa Kasse Figueiro Mastrangelli, Vânia Lopes Pinto, Simone Elias

**Affiliations:** I Universidade Federal de São Paulo. São Paulo, São Paulo, Brazil

**Keywords:** Body Image, Quality of Life, Breast Neoplasms, Self Concept, Public Health, Imagen Corporal, Calidad de Vida, Neoplasias de la Mama, Autoimagen, Salud Pública

## Abstract

**Objectives:**

to evaluate the quality of life and body image in women with breast cancer during chemotherapy, before and after interventions targeting body image.

**Methods:**

we conducted a prospective cohort study with 47 women, utilizing both qualitative and quantitative data analysis.

**Results:**

in the semi-structured interviews, 52.7% of the recommendations were deemed highly beneficial for the patients. After chemotherapy, 25.53% were dissatisfied with their body image, and 89.4% expressed concern about hair loss. Additionally, 97.9% of participants emphasized the importance of counseling tools in cancer treatment. We observed significant changes in body image scale scores and physical health (p<0.001), indicating an improvement in body image perception despite a decline in physical health when comparing pre- and post-chemotherapy results.

**Conclusions:**

the scarcity of Brazilian publications on body image is noted, underscoring the role of healthcare professionals in interdisciplinary approaches to enhance clinical practices.

## RESUMEN

**Objetivos:**

evaluar la calidad de vida y la imagen corporal en mujeres con cáncer de mama durante la quimioterapia, antes y después de la intervención en la imagen.

**Método:**

estudio de cohorte prospectivo con 47 mujeres, utilizando análisis cualitativo y cuantitativo de los datos.

**Resultados:**

el 52,7% de las orientaciones fueron altamente beneficiosas. Tras la quimioterapia, el 25,53% estaba insatisfecho con su imagen corporal y el 89,4% se preocupaba por la caída del cabello. El 97,9% destacó la relevancia de las herramientas de asesoramiento. Hubo cambios significativos en los puntajes de la escala de imagen corporal y la salud física (p<0,001), mostrando una mejora en la percepción corporal a pesar de la disminución de la salud física, pre- y post-quimioterapia.

**Conclusiones:**

se subraya la falta de publicaciones brasileñas sobre imagen corporal y la importancia de enfoques interdisciplinarios de los profesionales de la salud para mejorar las prácticas clínicas.

## INTRODUCTION

Breast cancer is the most common type of cancer globally, accounting for 11.7% of all cases. In Brazil, it is estimated that from 2023 to 2025, there will be 73,610 cases, with the Southeast region having the highest estimated risk at 84.46%^(1)^. Breast cancer is not a singular disease^(2)^ but rather a heterogeneous one, presenting multiple histopathological forms with different progressions and therapeutic responses^(3)^. This makes it one of the most feared cancers among women, as it not only affects their physical appearance but also significantly impacts their social relationships and self-image (SI), often leading to symptoms of anxiety and depression^(3)^.

All treatments can cause adverse effects, but chemotherapy (CT) is the one that most severely compromises the quality of life (QoL). Common symptoms include pain, inflammation, fatigue, nausea, vomiting, hair loss, and skin changes^(4)^. To counterbalance the impact of treatment, support networks—comprising family, partners, and friends—are critical. This is because women often turn to their social and spiritual/religious spheres for resources to cope with the condition^(4)^.

An enhanced perception of quality of life is also seen as a positive outcome, demonstrating that physical activity can shorten recovery time and promote well-being by reducing treatment side effects^(5)^. Each cancer patient must be evaluated individually, and if in good physical condition, it is recommended to encourage exercise to improve both self-esteem (SE) and QoL^(5)^.

Following a breast cancer diagnosis, women experience significant psychological and social changes, as the breast is regarded as a symbol of beauty, fertility, femininity, and health throughout all life stages^(6)^. In this context, patients undergo a significant process of restructuring their body image (BI) as they confront the disease and its treatments.

This prompted the present study, which aims to demonstrate how image consulting services can support women during and after cancer treatment. It is worth explaining that *image consulting* is understood as the construction of a visual identity based on analyses of each person’s body and facial biotypes, personal color palette, and behavior.

## OBJECTIVES

To assess the quality of life and body image in women with breast cancer during chemotherapy, before and after an intervention aimed at improving body image.

## METHODS

### Ethical aspects

The Research Ethics Committee of the Federal University of São Paulo (UNIFESP) approved the study. All participants signed the Informed Consent Form (ICF) in duplicate.

### Study design, period, and location

This is a prospective cohort study guided by the STROBE tool. We conducted it from 03/18/2016 to 05/24/2018 at the Outpatient Clinic of the Mastology Department in the Gynecology Division at São Paulo Hospital/University Hospital of the Federal University of São Paulo – UNIFESP.

### Study population; inclusion and exclusion criteria

The study invited women diagnosed with any malignant breast neoplasm, aged over 18, from any marital status, economic condition, and ethnicity, whether in menopause or not, and who had not yet begun chemotherapy treatment. Exclusion criteria included male patients and those who had already undergone chemotherapy. Of the 64 patients invited, 9 declined participation, and 8 passed away during data collection, resulting in a final sample of 47 women.

### Study protocol

The researcher invited patients while they waited in the clinic’s diagnostic consultation room on Fridays and for subsequent followup appointments on Mondays during the morning. The Body Image Scale (BIS) and the Functional Assessment of Cancer Therapy-General (FACT-G) were used at two time points: pre-chemotherapy (pre-CT) and post-chemotherapy (post-CT). The objective was to assess the patterns of quality of life (QoL) across physical, social/family, emotional, and functional domains, as well as to correlate body image (BI) with improvements in QoL among breast cancer patients.

The study considered the first point, T0 (before chemotherapy), where the researcher introduced the study. Upon agreeing to participate, the patient signed the Image Use Authorization Form (TAI), after which clinical data (CD) were collected using a form developed by the researcher. Subsequently, the FACT-G and BIS were administered. Personal and sociodemographic data from the Brazilian Institute of Geography and Statistics (IBGE) were also used. Additionally, a form with Image Consulting tools (ESE) was employed, including body type analysis, image cropping, style analysis references, and a personal coloring sheet featuring a four-family seasonal analysis. Lastly, personal contrast was evaluated.

### Data analysis and statistics

We described the quantitative characteristics of the participating women using summary measures such as means, standard deviations, medians, minimums, and maximums. In contrast, qualitative characteristics were presented in absolute and relative frequencies. The FACT-G scores were analyzed at different time points and compared using paired Wilcoxon tests. We assessed BIS questionnaire items over time and groups using generalized estimating equations (GEE) with a marginal Poisson distribution, identity link function, and exchangeable correlation matrix across evaluation time points. Additionally, Bonferroni multiple comparisons were conducted to verify differences across time points within each group and among the total sample. We performed all analyses using IBM-SPSS for Windows, version 20.0, with tabulations done in Microsoft Excel 2003, and tests were conducted considering a 5% significance level.

## RESULTS

The study included 47 women with an average age of 54. Of these, 34% were married, and 66% had no partner. Approximately 68% were already in menopause, which had started around the age of 47. Regarding education, 87% had completed elementary education, covering grades 1 through 5.

From the image consulting tools applied through the ESE, the patients reported the following about the consulting sessions during chemotherapy: 52.7% stated that the consultation “helped a lot”; 41% said it “helped somewhat,” and 5.1% said it “helped little”.

Regarding socioeconomic status, using data from the IBGE, 87.2% of participants were classified as belonging to class D and 12.8% to class C.

When analyzing clinical data, stage I was present in 14.9% of cases, stage II in 42.6%, stage III in 27.7%, and stage IV in 14.9%. Regarding treatment, 66% underwent neoadjuvant therapy, 38.1% had a total mastectomy, 61.9% had conservative surgery, and 31% underwent immediate reconstructive surgery.

After applying the BIS before chemotherapy, 27.65% were satisfied with their body image based on the BIS assessment, while 70.21% were dissatisfied either to a lesser or greater degree. These figures changed significantly after chemotherapy, with 4.25% reporting satisfaction and 95.74% showing dissatisfaction either to a lesser or greater degree, of which 53.19% experienced a lower level of dissatisfaction.

Regarding quality of life, 87.2% had higher FACT-G scores before chemotherapy, a percentage that decreased to 80.5% post-CT.

When asked if chemotherapy had altered their appearance, 89% reported changes, and 89.4% were bothered by hair loss. Additionally, 95.7% highlighted the importance of image consulting during treatment, 97.9% emphasized the importance of the guidance and information provided by the consultant, and 97.9% considered personal coloring analysis relevant.

Table 1 shows that only the BIS score and the physical domain of the FACT-G were statistically significant between the pre- and post-CT periods (p < 0.001), with the BIS score increasing significantly. In contrast, the physical well-being score decreased between the pre- and post-CT periods.

**Table 1:** Description of Body Image Scale and Functional Assessment of Cancer Therapy-General scores according to assessment time points and results of comparative tests between time points

Variable	Pre-CT	Post-CT	*p* value
BIS			
Mean ± SD	5.5 ± 6.5	11.6 ± 7.5	<0.001
Median (p25; p75)	3 (0; 8)	11 (7; 17)	
Physical			
Mean ± SD	21.3 ± 5.3	16.8 ± 6.7	<0.001
Median (p25; p75)	22 (18; 25)	16 (13; 23)	
Social/family			
Mean ± SD	18.9 ± 5.9	19.2 ± 5.5	0.535
Median (p25; p75)	20 (14; 24)	20 (17; 23)	
Emotional			
			0.654
Median (p25; p75)	18 (13; 20)	18 (12; 22)	
Functional			
Mean ± SD	16.9 ± 5.7	16.9 ± 6.5	0.935
Median (p25; p75)	18 (13; 22)	16 (13; 23)	
FACT – G			
Mean ± SD	73.8 ± 16.6	70.1 ± 17.1	0.186
Median (p25; p75)	76 (63; 83)	69 (57; 83)	

*Wilcoxon paired test; SD – standard deviation.*

Table 2 shows a statistically significant correlation between almost all FACT-G domains and the BIS at both evaluation time points (p < 0.05). The correlations between the BIS and FACT-G domains, as well as the total scale, were inverse (r < 0), indicating that higher BIS scores were associated with lower FACT-G domain scores. The observed correlations were direct (r > 0).

**Table 2:** Results of the correlations between the scales at each evaluation time point

Correlation		Pre-CT					Post-CT				
		BIS	Physical	Social/family	Emotional	Functional	BIS	Physical	Social/family	Emotional	Functional
Physical	r	-0.381					-0.475				
	*p*	0.008					0.001				
Social /family	r	-0.306	0.252				-0.391	0.327			
	*p*	0.037	0.087				0.007	0.025			
Emotional	r	-0.328	0.388	0.359			0.285	0.504	0.222		
	*p*	0.024	0.007	0.013			0.052	<0.001	0.133		
Functional	r	-0.515	0.331	0.613	0.617		-0.344	0.465	0.613	0.364	
	*p*	<0.001	0.023	<0.001	<0.001		0.018	0.001	<0.001	0.012	
FACT - G	r	-0.474	0.603	0.743	0.766	0.862	-0.504	0.778	0.699	0.658	0.824
	*p*	0.001	<0.001	<0.001	<0.001	<0.001	<0.001	<0.001	<0.001	<0.001	<0.001

*Spearman correlation.*

There were few statistically significant differences between BIS and FACT-G domains across the women’s characteristics categories. The pre-CT physical well-being score was statistically lower in women who had total mastectomy than those who had conservative surgery (p = 0.008). The pre-CT emotional well-being score was statistically lower in single women (p = 0.005), while the post-CT emotional well-being score was statistically higher in women from economic class D compared to class C2 (p = 0.029). Finally, the pre-CT functional well-being score and the pre-CT total FACT-G score were statistically lower in women who had undergone surgery (p = 0.020 and p = 0.013, respectively).

## DISCUSSION

Quality of life (QoL) and self-esteem (SE) are crucial topics in breast cancer treatment, as the removal of a breast can lead to significant psychological and physical impacts. Body image disturbance was indirectly linked to distress due to low self-compassion^(7)^. Mayara Ribeiro’s study provides preliminary evidence suggesting that self-compassion mediates the relationship between body image disturbance and psychological distress, highlighting a potentially protective effect of higher levels of self-compassion in women at risk for body image disturbances^(7)^. Breast cancer patients often experience dissatisfaction with their bodies, with various emotions arising from the disease’s diagnosis and treatment. A study reveals patient testimonials about how they feel regarding their body image — “emotional scar”^(7)^. Notably, our study, conducted at the Mastology Department of UNIFESP, pioneered the provision of OncoImage services, which contribute to improving patients’ SE and QoL.

When assessing the quality of life, we observed statistically significant changes between pre-CT and post-CT assessments in both the BIS score and the physical domain of the FACT-G (p < 0.001). Specifically, the BIS score showed a statistically significant increase, while the physical well-being score declined from pre- to post-chemotherapy. This result suggests that despite the OncoImage interventions employed during the study, patients still reported dissatisfaction with their body image during chemotherapy treatments.

It is essential to examine these women’s daily routines, particularly regarding the reconstruction of body image, to empower healthcare professionals to promote well-being^(8)^. A systematic review conducted in 2017, with a sample of 82% breast cancer patients and 18% from mixed populations (including gynecological, gastrointestinal, genitourinary, and hematologic cancers), evaluated the measurement properties of the BIS. Evidence regarding the internal consistency, structural validity, and reliability of the questionnaire was rated as sufficient^(8)^. Another study by Rhondali cited that 58% of young, low-income patients experienced greater dissatisfaction with their body image^(9)^.

Other studies have focused on investigating the needs of breast cancer survivors, aiming to ensure longevity with QoL for these patients^(10,11,12)^. Multidisciplinary teamwork aimed at non-medical treatment is essential, as is the implementation of comprehensive psychosocial support programs for both patients and their families throughout the disease’s trajectory; furthermore, changes in SE, along with OncoImage (which provides tools to support patients coping with hair loss during chemotherapy), are crucial in enhancing these patients’ SE^(10,11,12)^.

In this qualitative study, which included women aged 18 to 40, decreased SE in the younger group was a significant aspect associated with hair loss, breast removal, and a lack of independence. Issues such as sexuality and self-perception are fundamental for these women but often receive little attention in medical care, which focuses on the disease. Therefore, improving guidance in these areas is vital^(13)^.

Self-esteem can be characterized as an individual’s positive feeling about themselves, crucial in social interactions^(14)^. Cancer patients generally experience various changes in SE, from accepting the diagnosis to coping with treatments, which can negatively impact their body image and well-being^(14)^.

Above all, each individual diagnosed with cancer must be evaluated individually. If in good physical condition, physical activity should not only be encouraged but also promoted as a way to improve SE and QoL^(5)^. A 2020 study indicates that breast cancer patients’ QoL has improved over the past decade^(15)^. This improve -ment is attributed to several simple yet effective approaches, such as physical exercises and psychosocial interventions^(15)^.

In our study, 27.65% of patients were satisfied with their image after the BIS application and before chemotherapy, and 70.21% were dissatisfied either to a lesser or greater degree. These data significantly changed post-CT, with 42.55% of patients satisfied and 95.74% dissatisfied either to a lesser or greater degree, with 53.19% reporting mild dissatisfaction.

A systematic review conducted in 2018 demonstrates that altered body appearance following cancer treatment can be accompanied by feelings of shame, negative SE, or social avoidance^(8)^. This finding supports our results, which show that this negativity is linked to dissatisfaction with body appearance.

In 2020, researchers conducted an exploratory study in a major hospital in Rio de Janeiro using a quantitative and qualitative approach. They used the BIS to interview 60 patients in the oncology sector at the chemotherapy outpatient clinic. It was observed that SE can be recognized through verbal reports, behaviors, and attitudes, such as the tendency to stay isolated at home, reduced health care, and complaints about their living environment, indicating low SE^(16)^. Lack of SE can affect not only how a woman perceives herself but also how she views the world around her, leading to decreased interest in social and personal activities^(16)^. In this regard, the importance of family, social, and religious support is undeniable for women with breast cancer, as it provides valuable contributions to coping throughout all stages of treatment^(16)^. This outcome aligns with our research, where 80% of patients presented with symptoms predominantly related to body image disturbances. Similarly, another study involving 100 women with breast cancer using the BIS demonstrated that they experience low SE associated with body image disturbances^(17)^.

OncoImage services can play a particularly important role in improving breast cancer patients’ SE by addressing modifiable factors that can elevate SE. Given this fact, Pena emphasizes that it is essential to establish a self-care program for body image throughout life, especially for women undergoing treatment^(18)^. As demonstrated in our study, 93% of women accepted image consulting guidance, with 57.44% reporting satisfaction with their image after chemotherapy.

The realm of personal appearance is defined by aesthetics centered on the individual; however, there have been similar practices to image consulting throughout history, dating back to the time of Marie Antoinette^(5)^. In the past, image consultants were included among fashion stylists’ recommendations on clients’ clothing, accessories, and conduct^(5)^. Since ancient times, personal appearance has been crucial in social interaction, highlighting the importance of image and perception^(5)^. When meeting someone for the first time, an initial rapid assessment occurs. This assessment is not only verbal but also visual, in which factors like appearance, body language, and facial expressions have a significant influence on the impression we leave^(19)^. These reactions can result in either positive or negative impressions when encountering someone for the first time. Given the information overload in the fashion industry, consumers often choose clothing impulsively, which can lead to misconceptions about their personal image and even harm them in various situations^(19)^.

Pena notes that some patients undergoing physical changes may have their body image and SE affected, which can make treatment more challenging and slower; they may also be vulnerable to negative reactions from others^(18)^. Maintaining a positive image during treatment can help reduce concerns about how people perceive them. Certain precautions are essential, as these treatments entail various side effects, leading to both physical (related to body image) and emotional changes (pertaining to attitudes, personality, and abilities). Cancer treatments can cause alterations to a patient’s image, such as changes in skin tone, thinning of eyelashes and eyebrows, hair loss, fatigue, dark circles, and a lack of motivation to maintain their appearance^(5)^.

OncoImage professionals are those who address patients’ body image and personal image during cancer treatment^(20)^. Presently, an organization called ABIHPEC (Brazilian Association of Personal Hygiene, Perfumery, and Cosmetics Industry) works to promote SE, beauty, and well-being for breast cancer patients in some hospitals^(20)^.

Integrative Oncology (IO), part of Integrative Medicine (IM), combines evidence-based complementary practices with conventional medicine. It encompasses biologically-based approaches, mind-body techniques, body manipulation, energy therapies, and traditional medical systems^(21)^. The understanding of IO is well-established at leading cancer research and treatment centers in North America, with a significant amount of literature available in this field. When used alongside conventional treatment, integrative approaches can enhance effectiveness and alleviate adverse symptoms of cancer^(21)^.

Enhancing care for women with breast cancer extends beyond physical treatment, as the psychological implications directly affect their QoL. It is, therefore, crucial for healthcare professionals to recognize these implications and establish guidelines to ensure the well-being of these patients^(22)^.

Women who survive breast cancer need to receive information and expand their knowledge about the disease and its consequences to facilitate adapting to their transformed bodies and expedite their return to daily routines. This can strengthen their self-confidence and self-perception, enabling their social reintegration and improving their quality of life^(23)^.

### Study limitations

This study is limited by the small sample size, which was influenced by the duration of treatment. Additionally, the analysis of results was hindered by the lack of published studies using the same method for evaluating body image.

### Contributions to the field of Nursing, Health, or Public Policy

Our study contributes to nursing teams caring for this population by offering guidance on using image consulting tools and highlighting their potential impact on the quality of life for these women.

## CONCLUSIONS

We concluded that patients’ quality of life decreased during chemotherapy, affecting their self-esteem. We emphasize the importance of image consulting during treatment, with 97.4% of patients positively evaluating these services. Investigating how cultural symbols influence body image perception is crucial, especially when physical changes occur due to the disease and treatments. Identifying discourses centered around alternative beauty standards may reveal diverse meanings attributed to the female body, challenging the dominance of traditional cultural norms.

In this study, we found a need for interdisciplinary approaches and care to improve clinical practices. Additionally, we highlight the scarcity of Brazilian studies on body image in women with breast cancer, underlining the importance of research that explores these women’s experiences within the Brazilian sociocultural context.
