# Supplementary material for: The impact on body image and quality of life in breast cancer patients
Source: Rev Bras Enferm. 2025 Mar 10;78(1):e20240207. doi: 10.1590/0034-7167-2024-0207 (PMC11893061; doi:10.1590/0034-7167-2024-0207)
Supplement: Translation

# Translation: O impacto na imagem corporal e qualidade de vida das pacientes com câncer de mama

**DOI:** 10.1590/0034-7167-2024-0207pt

**Authors:** Andressa Kasse Figueiro Mastrangelli, Vânia Lopes Pinto, Simone Elias

**Affiliations:** I Universidade Federal de São Paulo. São Paulo, São Paulo, Brasil

**Keywords:** Imagem Corporal, Qualidade de Vida, Neoplasias da Mama, Autoimagem, Saúde Pública

## Abstract

**Objetivos:**

avaliar a qualidade de vida e a imagem corporal em mulheres com câncer de mama durante a quimioterapia, antes e depois da intervenção na imagem.

**Métodos:**

estudo de coorte prospectivo com 47 mulheres, usando análise dos dados qualitativa e quantitativa.

**Resultados:**

nas entrevistas semiestruturadas, 52,7% das orientações foram altamente benéficas para as pacientes. Após a quimioterapia, 25,53% estavam insatisfeitas com sua imagem corporal; e 89,4%, preocupadas com a queda de cabelo. A importância dos instrumentos de aconselhamento no tratamento do câncer foi destacada por 97,9%. Mudanças significantes nos escores da escala de imagem corporal e na saúde física foram observadas (p<0,001), indicando melhora na percepção corporal, apesar da redução na saúde física, considerando antes e depois da quimioterapia.

**Conclusões:**

ressalta-se a escassez de publicações brasileiras sobre imagem corporal e destaca-se o papel dos profissionais de saúde em abordagens interdisciplinares para melhorar as práticas clínicas.

## INTRODUÇÃO

O câncer de mama é o tipo mais incidente no mundo, com 11,7% do total de casos. No Brasil, para o triênio de 2023 a 2025, estimam-se 73.610 casos, sendo que a região Sudeste tem o maior risco estimado, 84,46%^(1)^. Esse câncer não é uma doença única^(2)^, e sim heterogênea, apresentando múltiplas formas histopatológicas bem como diferentes evoluções e respostas terapêuticas^(3)^. Por causa disso, é um dos mais assustadores entre as mulheres, por não apenas comprometer o seu corpo, mas principalmente impactar as relações sociais e a sua autoimagem (AI), podendo resultar em sintomas de ansiedade e depressão^(3)^.

Todos os tratamentos podem gerar efeitos adversos. Dentre eles, a quimioterapia (QT) é o que compromete mais severamente a qualidade de vida. Os sintomas frequentemente relacionados incluem dor, inflamação, cansaço, náusea, vômito, queda de cabelo e alterações cutâneas^(4)^.

Para contrabalançar o impacto do tratamento, é essencial a rede de suporte, que envolve familiares, parceiros e amigos. Isso porque as mulheres buscam no núcleo social e na esfera religiosa/ espiritual recursos para enfrentar a condição^(4)^.

A percepção de uma qualidade de vida (QV) aprimorada também é vista como um efeito positivo, demonstrando que a atividade física pode encurtar o tempo de recuperação do tratamento contra o câncer e proporcionar uma sensação de bem-estar aos pacientes por meio da diminuição dos efeitos colaterais^(5)^. Cada paciente com câncer deve ser avaliado individualmente; se estiver em boas condições físicas, é recomendável estimular a prática de exercícios para melhorar tanto a autoestima (AE) quanto a QV^(5)^.

Após o diagnóstico de câncer de mama, as mulheres experimentam mudanças psicológicas e sociais, já que a mama é vista como um símbolo de beleza, fertilidade, feminilidade e saúde em todas as fases da vida^(6)^. Nesse cenário, as pacientes enfrentam um significativo processo de reestruturação da sua imagem corporal (IC) quando confrontadas com a doença e seus tratamentos.

Esse fato nos levou à realização deste estudo com o objetivo de mostrar o quanto os serviços de consultoria de imagem podem auxiliar as mulheres durante e após o tratamento oncológico. Vale dizer que se entende como “consultoria de imagem” a construção de uma identidade visual com base em análises dos biótipos corporal e facial, da coloração pessoal e do comportamento de cada pessoa.

## OBJETIVOS

Avaliar a qualidade de vida e a imagem corporal em mulheres com câncer de mama durante a quimioterapia, antes e depois da intervenção na imagem.

## MÉTODOS

### Aspectos éticos

O estudo foi aprovado pelo Comitê de Ética em Pesquisa da Universidade Federal de São Paulo (UNIFESP). Todas as participantes assinaram o Termo de Consentimento Livre e Esclarecido (TCLE) em duas vias.

### Desenho, período e local do estudo

Trata-se de um estudo de coorte prospectivo, norteado pela ferramenta STROBE. Foi realizado entre 18/03/2016 e 24/05/2018, no Ambulatório da Disciplina de Mastologia do Departamento de Ginecologia do Hospital São Paulo/Hospital Universitário da Universidade Federal de São Paulo – UNIFESP.

### População do estudo; critérios de inclusão e exclusão

Para constituir a amostra deste estudo, foram convidadas pacientes diagnosticadas com qualquer neoplasia maligna da mama, com idade superior a 18 anos, em qualquer estado civil e condição econômica, pertencentes a qualquer etnia, na menopausa ou não e que ainda não tivessem iniciado o tratamento de quimioterapia. Os critérios de exclusão foram sexo masculino e pacientes que já haviam realizado o tratamento quimioterápico. Foram convidadas a participar da pesquisa 64 pacientes, das quais 9 recusaram e 8 faleceram no decorrer da coleta, resultando em uma amostra final de 47 mulheres.

### Protocolo do estudo

Essas pacientes foram convidadas pela pesquisadora quando estavam na sala de espera do ambulatório para a consulta diagnóstica, às sextas-feiras; e nos atendimentos subsequentes de acompanhamento para o estudo, às segundas-feiras, no período da manhã.

Foram utilizados a *Body Image Scale* (BIS), o *Functional Assessment of Cancer Therapy-General* (FACT-G), em dois momentos/tempos: pré-QT e pós-QT. O intuito foi avaliar os padrões de QV nos domínios físico, social/familiar, emocional e funcional, bem como associar a IC com a melhora na QV de mulheres com câncer de mama.

No estudo, foi considerado o primeiro momento T0 (antes da QT), em que a pesquisadora informava sobre o estudo. A paciente, ao aceitar participar, assinava o termo de Autorização de Uso de Imagem (TAI), depois eram coletados os dados clínicos (DC) por meio de um formulário criado pela pesquisadora. Na sequência, eram aplicados o FACT-G e a BIS. Foram usados também dados do Instituto Brasileiro de Geografia e Estatística (IBGE) quanto aos dados pessoais e sociodemográficos. Ainda, utilizou-se um formulário desenvolvido com as ferramentas da Consultoria de Imagem (ESE) compreendendo análise do biótipo (corpo), recorte de imagem e referências para a análise de estilo, além de ficha técnica de coloração pessoal, composta da análise sazonal de quatro famílias. Por último, foi avaliado o contraste pessoal.

### Análise dos dados e estatística

As características quantitativas das mulheres participantes foram descritas com uso de medidas de resumo, como médias, desvios-padrão, medianas, mínimos e máximos, enquanto as características qualitativas foram apresentadas em frequências absolutas e relativas. Os escores dos questionários FACT-G foram analisados em diferentes momentos e comparados usando testes Wilcoxon pareados. Os itens do questionário BIS foram avaliados ao longo dos momentos e grupos mediante equações de estimação generalizadas (EEG) com distribuição marginal Poisson, função de ligação identidade e matriz de correlações permutáveis entre os momentos de avaliação para todas as análises. Adicionalmente, foram realizadas comparações múltiplas de Bonferroni para verificar diferenças entre os momentos de avaliação em cada grupo e no total das mulheres. Todas as análises foram conduzidas utilizando o software IBM-SPSS for Windows - versão 20.0, com tabulações feitas no Microsoft Excel 2003, e os testes foram conduzidos considerando o nível de significância de 5%.

## RESULTADOS

A população do estudo foi de 47 mulheres com uma média de idade de 54 anos, das quais 34% eram casadas, e 66% não tinham companheiro. Cerca de 68% já estavam na menopausa e iniciaram essa fase em torno de 47 anos. Quanto à escolaridade, 87% tinham o ensino fundamental I, que vai do 1º ao 5º ano.

Nos resultados da aplicação das ferramentas de consultoria de imagem por meio do ESE, as orientações da consultoria durante o tratamento de QT foram relatadas pelas pacientes da seguinte maneira: 52,7% disseram que a consultoria “ajudou muito”; para 41%, “ajudou mais ou menos”; e para 5,1%, “ajudou pouco”.

Quando aplicado o formulário com os dados do IBGE para verificar questões socioeconômicas, 87,2% pertenciam à classe D; e 12,8%, à classe C.

Analisando os dados clínicos, o estadiamento I esteve presente em 14,9% dos casos; o II, em 42,6%; o III, em 27,7%; e o IV, em 14,9%. No tocante ao tratamento, 66% realizaram neoadjuvância; 38,1% foram submetidas à mastectomia total; 61,9%, à cirurgia conservadora; e 31%, à cirurgia reconstrutiva imediata.

Após a aplicação do BIS antes da QT, 27,65% estavam satisfeitas com a imagem; e 70,21%, insatisfeitas em menor ou alto grau. Esses dados se modificaram significativamente depois da QT, quando 4,25% se apresentaram satisfeitas, e 95,74%, insatisfeitas em menor ou alto grau, sendo 53,19% em menor grau.

Em relação à QV, 87,2% apresentaram maior índice do FACT-G antes da QT, e essa porcentagem foi reduzida para 80,5% após a QT.

Quando questionadas sobre a QT ter modificado a sua aparência, 89% relataram modificação, 89,4% se incomodaram com a queda do cabelo, 95,7% relataram a importância da consultoria de imagem durante o tratamento, 97,9% enfatizaram a importância das orientações e informações realizadas pelo profissional consultor, e 97,9% consideraram relevante a compreensão da coloração pessoal.

A Tabela 1 apresenta o resultado em que apenas o escore do BIS e o domínio físico do FACT-G foram estatisticamente significantes entre o momento pré-QT e o pós-QT (p < 0,001), sendo que o escore BIS aumentou estatisticamente e o escore de bem-estar físico diminuiu entre o pré-QT e o pós-QT.

**Tabela 1:** Descrição dos escores Body Image Scale e Functional Assessment of Cancer Therapy-General segundo momentos de avaliação e resultado dos testes comparativos entre os momentos

Variável	Pré-QT	Pós-QT	Valor de p
BIS			
Média ± DP	5,5 ± 6,5	11,6 ± 7,5	<0,001
Mediana (p25; p75)	3 (0; 8)	11 (7; 17)	
Físico			
Média ± DP	21,3 ± 5,3	16,8 ± 6,7	<0,001
Mediana (p25; p75)	22 (18; 25)	16 (13; 23)	
Social/familiar			
Média ± DP	18,9 ± 5,9	19,2 ± 5,5	0,535
Mediana (p25; p75)	20 (14; 24)	20 (17; 23)	
Emocional			
Média ± DP	16,7 ± 4,9	17,2 ± 5,1	0,654
Mediana (p25; p75)	18 (13; 20)	18 (12; 22)	
Funcional			
Média ± DP	16,9 ± 5,7	16,9 ± 6,5	0,935
Mediana (p25; p75)	18 (13; 22)	16 (13; 23)	
FACT – G			
Média ± DP	73,8 ± 16,6	70,1 ± 17,1	0,186
Mediana (p25; p75)	76 (63; 83)	69 (57; 83)	

*Teste de Wilcoxon pareado; DP – desvio-padrão.*

A Tabela 2 mostra que houve correlação estatisticamente significante entre quase todos os domínios do FACT-G e com o BIS em ambos os momentos de avaliação (p < 0,05). As correlações entre o BIS e os domínios do FACT-G bem como com o total da escala foram inversas (r < 0), ou seja, quanto maior o escore BIS, menores os escores dos domínios do FACT-G. As correlações observadas foram diretas (r > 0).

**Tabela 2:** Resultado das correlações entre as escalas em cada momento de avaliação

Correlação		Pré-QT					Pós-QT				
		BIS	Físico	Social/familiar	Emocional	Funcional	BIS	Físico	Social/familiar	Emocional	Funcional
Físico	r	-0,381					-0,475				
	*p*	0,008					0,001				
Social/familiar	r	-0,306	0,252				-0,391	0,327			
	*p*	0,037	0,087				0,007	0,025			
Emocional	r	-0,328	0,388	0,359			0,285	0,504	0,222		
	*p*	0,024	0,007	0,013			0,052	<0,001	0,133		
Funcional	r	-0,515	0,331	0,613	0,617		-0,344	0,465	0,613	0,364	
	*p*	<0,001	0,023	<0,001	<0,001		0,018	0,001	<0,001	0,012	
FACT - G	r	-0,474	0,603	0,743	0,766	0,862	-0,504	0,778	0,699	0,658	0,824
	*p*	0,001	<0,001	<0,001	<0,001	<0,001	<0,001	<0,001	<0,001	<0,001	<0,001

*Correlação de Spearman.*

Houve poucas diferenças estatisticamente significantes nas escalas BIS e domínios do FACT-G entre as categorias das características das mulheres. O escore de bem-estar físico pré-QT foi estatisticamente menor nas mulheres que fizeram mastectomia total do que nas que fizeram cirurgia conservadora (p = 0,008). O escore de bem-estar emocional pré-QT foi estatisticamente menor nas mulheres solteiras (p = 0,005), enquanto o escore de bem-estar emocional pós-QT foi estatisticamente maior nas mulheres de classe econômica D do que C2 (p = 0,029). Por fim, o escore de bem-estar funcional pré-QT e o FACT-G total pré-QT foram estatisticamente menores nas mulheres que fizeram cirurgia (p = 0,020 e p = 0,013, respectivamente).

## DISCUSSÃO

A QV e a AE são pautas importantes quando o assunto é câncer de mama, visto que a retirada da mama pode causar grande impacto psicológico e físico. A perturbação da IC foi indiretamente associada à angústia por meio da baixa autocompaixão^(7)^. No estudo de Mayara Ribeiro, são fornecidas evidências preliminares de um papel mediador da autocompaixão entre o distúrbio da IC e o sofrimento psicológico, sugerindo um efeito potencialmente protetor de níveis mais altos de autocompaixão para mulheres em risco de sofrer distúrbios na IC^(7)^. As pacientes com câncer de mama apresentam insatisfação com o corpo: vários sentimentos surgem desde o diagnóstico da doença e durante o tratamento. Estudo revela depoimentos de pacientes sobre como eles se sentem com a sua imagem — “cicatriz emocional”^(7)^. Vale ressaltar que a nossa pesquisa, realizada no departamento de Mastologia da Unifesp, foi pioneira com o atendimento de Oncoimagem, que auxilia na AE e QV das pacientes.

Mensurando a qualidade de vida, no domínio físico, observamos que o escore do BIS e o domínio físico do FACT-G apresentam alteração estatisticamente significante entre o momento pré-QT e o pós-QT (p < 0,001), sendo que o escore do BIS aumentou estatisticamente e o escore do bem-estar físico diminuiu (do pré-QT para o pós-QT). Esse resultado indica que, com os tratamentos de QT e atendimentos/ferramentas de oncoimagem utilizadas durante a pesquisa, as pacientes mostraram insatisfação com a imagem.

É essencial examinar a rotina dessas mulheres, especialmente na reconstrução da IC, a fim de fornecer informações para capacitar profissionais de saúde na promoção do bem-estar^(8)^. Uma revisão sistemática^(8)^, realizada em 2017, apresenta uma amostra composta de 82% dos pacientes com câncer de mama e 18% de uma população mista (incluindo câncer ginecológico, gastrointestinal, geniturinário e hematológico). Foram avaliadas as propriedades de medição do BIS, em que as evidências sobre consistência interna, validade estrutural e confiabilidade desse questionário foram classificadas como suficientes^(8)^. Em outra pesquisa, o autor Rhondali cita que 58% das pacientes jovens e pobres tiveram maior insatisfação com a IC^(9)^.

Outros estudos têm se preocupado com a investigação das necessidades dos sobreviventes de câncer de mama, visando à longevidade com QV para as pacientes^(10,11,12)^. Certamente, trabalhar com uma equipe multidisciplinar direcionada para o tratamento não medicamentoso é essencial; assim como a implementação de um programa abrangente de suporte psicossocial para pacientes e seus familiares durante o enfrentamento da doença; além disso, alterações na AE, juntamente com a oncoimagem (que traz ferramentas de apoio para o surgimento de alopecia nas pacientes durante o tratamento de QT), são fundamentais para que a AE dessas pacientes possa ser aumentada^(10,11,12)^.

Nesse estudo qualitativo, envolvendo mulheres de 18 a 40 anos, destaca-se a diminuição da autoestima no grupo jovem como um aspecto significativo associado à queda de cabelo, remoção da mama e falta de independência. Temas como sexualidade e percepção pessoal são fundamentais para essas mulheres, mas frequentemente recebem pouca atenção na assistência médica, que focaliza a doença. Por isso, é vital melhorar a orientação nessas áreas^(13)^.

A AE pode ser caracterizada como o sentimento positivo que uma pessoa tem em relação a si mesma, desempenhando um papel crucial em suas interações sociais^(14)^. Geralmente pacientes com câncer enfrentam diversas mudanças na AE, desde a aceitação do diagnóstico até a conformidade com os tratamentos, que podem afetar negativamente sua AI e bem-estar^(14)^.

Acima de tudo, cada indivíduo diagnosticado com câncer precisa ser avaliado de forma personalizada; e, se estiver em boa condição física, a realização de atividades físicas não apenas pode, mas deve ser encorajada, visando aprimorar a AE e a QV^(5)^. Um estudo realizado em 2020 indica que a QV de pacientes com câncer de mama aumentou ao longo dos últimos dez anos^(15)^. Isso porque diversas abordagens simples, porém eficazes, como exercícios físicos e intervenções psicossociais, foram utilizadas para alcançar esses resultados^(15)^.

No nosso trabalho, após a aplicação do BIS e antes da QT, 27,65% das pacientes estavam satisfeitas com a imagem, e 70,21% estavam insatisfeitas em menor ou alto grau. Esses dados se modificaram significativamente depois da QT, quando 42,55% das pacientes se apresentaram satisfeitas, e 95,74%, insatisfeitas em menor ou alto grau, sendo 53,19% em menor grau.

Uma revisão sistemática realizada em 2018 demonstra que a aparência corporal alterada após o tratamento do câncer pode ser acompanhada de sentimentos de vergonha, AE negativa ou do ato de esquivar-se socialmente de compromissos^(8)^. Tal achado corrobora o nosso resultado, que traz essa negatividade relacionada à insatisfação com a aparência corporal.

Em 2020, num hospital de grande porte do Rio de Janeiro, ocorreu uma pesquisa exploratória com abordagem quantiqualitativa. Foi usada a escala de investimento corporal (BIS) para entrevistar 60 pacientes no setor de oncologia, no ambulatório de QT. Notou-se que a AE pode ser reconhecida por meio de relatos verbais, comportamentos e atitudes, como a tendência ao isolamento em casa, a redução dos cuidados com a saúde e as queixas sobre o ambiente em que vivem, sugerindo uma baixa AE^(16)^. Dessa forma, a falta de AE pode afetar não apenas a visão que a mulher tem de si mesma, mas também a percepção do mundo ao seu redor, resultando na diminuição do interesse em atividades sociais e pessoais^(16)^. Nesse sentido, a relevância do suporte familiar/social e religioso é inegável para mulheres com câncer de mama, oferecendo valiosas contribuições para o enfrentamento das pacientes em todos os estágios do tratamento^(16)^. Esse resultado está em consonância com o de nossa pesquisa, em que 80% das pacientes apresentaram como maior sintoma perturbações relacionadas à sua AI. Similarmente, outra pesquisa envolvendo 100 mulheres com câncer de mama e utilizando o BIS demonstrou que elas experienciam baixa AE ligada à IC^(17)^.

Os serviços de oncoimagem podem desempenhar um papel particularmente importante no aumento da AE das pacientes com câncer de mama, interferindo nos fatores modificáveis para a elevação da AE. Considerando esse fato, Pena afirma que se torna essencial estabelecer um programa de prática de autocuidado na AI ao longo da vida, principalmente para as mulheres em tratamento^(18)^. Como apresentado em nosso estudo, 93% das mulheres aceitaram as orientações da consultoria de imagem, das quais 57,44% obtiveram satisfação com a imagem pós-QT.

A esfera da aparência pessoal é definida pelo trabalho estético centrado no indivíduo; ao longo da história da moda, já houve atividades semelhantes à consultoria de imagem desde os tempos de Maria Antonieta^(5)^. Os serviços de conselheiro estavam incluídos nas recomendações de estilistas sobre vestuário, acessórios e conduta dos clientes^(5)^. Desde tempos antigos, a aparência pessoal desempenha um papel crucial na interação social, portanto fica evidente a importância da imagem e da percepção^(5)^. No primeiro encontro com alguém, ocorre uma rápida avaliação inicial da pessoa. É estabelecida uma conexão não apenas verbal, mas também visual, na qual fatores como aparência, linguagem corporal e expressões faciais exercem forte influência na impressão que deixamos^(19)^. Essas reações podem resultar em impressões positivas ou negativas ao se encontrar alguém pela primeira vez. Diante da sobrecarga de informações na indústria da moda, o consumidor muitas vezes opta por suas roupas de maneira impulsiva, o que pode levar a interpretações equivocadas sobre sua imagem pessoal e até mesmo prejudicar o indivíduo em várias situações^(19)^.

Pena cita que algumas pacientes que passam por mudanças físicas podem ter a AI e a AE afetadas, o que torna o tratamento mais difícil e lento; além disso, elas ficam muito vulneráveis a reações negativas de outras pessoas^(18)^. Manter uma imagem positiva durante o estágio pode ajudar a diminuir a preocupação com a maneira como as pessoas as veem. Certos cuidados são importantes, uma vez que tais procedimentos acarretam vários efeitos colaterais, trazendo mudanças físicas (ligadas à imagem do corpo) e emocionais (referentes a atitudes, personalidade e habilidades). Os tratamentos oncológicos podem provocar alterações na imagem da paciente, como mudança no tom de pele, escasseamento de cílios e de sobrancelhas, queda de cabelo, cansaço, olheiras, desânimo e falta de vontade de se arrumar^(5)^.

Os profissionais de oncoimagem são aqueles que tratam da IC e pessoal do paciente durante o tratamento oncológico^(20)^. Atualmente existe uma instituição chamada ABIHPEC (Associação Brasileira da Indústria de Higiene Pessoal, Perfumaria e Cosmética), cujo trabalho é promover, em alguns hospitais, a AE, a beleza e o bem-estar das pacientes diagnosticadas com câncer de mama^(20)^.

A Oncologia Integrativa (OI), uma parte da Medicina Integrativa (MI), combina práticas complementares baseadas em evidências favoráveis com a medicina tradicional, abrangendo técnicas apoiadas na biologia, técnicas mente-corpo, manipulação corporal, terapias energéticas e sistemas médicos tradicionais^(21)^. O entendimento da OI está firmemente estabelecido nos principais centros de pesquisa e assistência ao câncer na América do Norte, e há uma quantidade considerável de literatura disponível sobre esse novo campo. Quando usadas em conjunto com o tratamento convencional, as abordagens integrativas podem promover a eficácia e aliviar os sintomas adversos do câncer^(21)^.

Aprimorar o cuidado oferecido às mulheres com câncer de mama vai além do tratamento físico, visto que as repercussões psicológicas têm relação direta com a qualidade de vida delas; portanto, é crucial que o profissional de saúde reconheça tais implicações e estabeleça condutas para assegurar o bem-estar dessas pacientes^(22)^.

As mulheres que sobreviveram ao câncer de mama precisam receber esclarecimentos e ampliar seu conhecimento sobre a enfermidade e suas consequências, visando facilitar a adaptação ao corpo transformado e acelerar a retomada das rotinas diárias. Isso pode fortalecer a autoconfiança e a percepção de si mesmas, possibilitando sua reintegração social e um aumento da qualidade de vida^(23)^.

### Limitações do estudo

Este estudo tem uma limitação relacionada ao tamanho da amostra, que foi influenciado pela duração do tratamento. Além disso, a análise dos resultados foi dificultada pela falta de publicações que utilizam o mesmo método de avaliação da imagem corporal.

### Contribuições para a área da Enfermagem, Saúde ou Políticas Públicas

Nosso estudo contribui com a equipe de enfermagem que atende essa população e pode fornecer orientações sobre as ferramentas da consultoria de imagem e sobre o quanto isso pode influenciar a qualidade de vida dessas mulheres.

## CONCLUSÕES

Concluímos que a qualidade de vida das pacientes diminuiu durante a quimioterapia, afetando sua autoestima. Destacamos a importância da consultoria de imagem durante o tratamento, com 97,4% das pacientes avaliando positivamente as ferramentas desse serviço. É crucial investigar como os símbolos culturais influenciam a percepção da IC, especialmente quando alterações físicas ocorrem devido à doença e tratamentos. A identificação de discursos pautados em padrões de beleza alternativos pode indicar uma diversidade de significados atribuídos ao corpo feminino, questionando a hegemonia desses padrões culturais.

Neste estudo, constatamos a necessidade de abordagens e atendimentos interdisciplinares para melhorar as práticas clínicas. Ressaltamos também a escassez de estudos brasileiros sobre IC em mulheres com câncer de mama, evidenciando a importância de pesquisas que explorem a experiência dessas mulheres no contexto sociocultural brasileiro.
